# Association of working conditions with employer’s cost of presenteeism and sickness absence among young and early midlife municipal employees: a register-linked study

**DOI:** 10.1186/s12995-026-00534-4

**Published:** 2026-10-26

**Authors:** Anna C. Svärd, Mari-Liis Kalima, Kimmo Vänni, Eira Roos, Riitta Luoto, Tea Lallukka

**Affiliations:** 1 Department of Public Health, Faculty of Medicine, University of Helsinki, PO BOX 20 (Tukholmankatu 8 B), Helsinki, FIN-00014 Finland; 2 HAMK Tech, Häme University of Applied Sciences, Hämeenlinna, Finland

**Keywords:** Cohort study, Disability, Sick leave, Work strain

## Abstract

**Background:**

Working conditions are associated with sickness absence (SA), but less is known about their associations with presenteeism and, more specifically, direct cost of presenteeism and SA. We examined how working conditions are associated with these.

**Methods:**

We prospectively linked the Helsinki Health Study 2017 survey on workplace atmosphere, workplace bullying, social support, organizational justice, work-family conflict, and physical working conditions with employer-provided salary and sickness absence data during a one-year follow-up among 19–39-year-old employees (*n* = 3,069, women 78%). We estimated presenteeism days using the baseline Work Ability Score (WAS, work ability 0–10) and WAS-related discount factors. Employer’s cost of presenteeism and sickness absence of first 1–10 days (100% employer’s cost) and subsequent 11–60 days (30% employer’s cost) were calculated with a two-part model, adjusting for marital status and education.

**Results:**

Mean annual employer’s cost of presenteeism, sickness absence of 1–10 days, and 11–60 days were €2,394, €2,229, and €541 for women, and €2,245, €1,422, and €306 for men, respectively. Overall, presenteeism costs were up to twice as high among participants reporting psychosocially demanding work, but smaller for physical working conditions. Especially those with high work-family conflicts (women: €3,108, 95% CI €2,762–3,454; men €2,701, 95% CI €1,993–3,409) and poor workplace atmosphere (women: €2,203, 95% CI €1,443–2,962; men €3,136, 95% CI €1,219–5,053) had higher presenteeism cost compared to those without. The differences in the cost of the 10 first sickness absence days were similar but smaller than those for presenteeism, while differences in 11–60 days cost could not be statistically confirmed. Adjusting for education and marital status marginally contributed to the findings.

**Conclusion:**

Psychosocial working conditions were associated with higher employer’s cost of presenteeism and sickness absence, suggesting that improving them may reduce costs and support productivity, work ability, and broader economic outcomes.

## Background

Safe and healthy working conditions are a global long-term goal, and continue to be a key priority for modern societies [1, 2]. For example, employers in the European Union have a legal responsibility to ensure a safe and healthy working environment, and globally, promotingsafe and secure working environments for all is included in Goal 8 of the UN 2030 Agenda [1]. While improvements have especially been made in physical safety and the reduction of occupational hazards, mental and emotional demands have grown as work has become increasingly cognitively and psychologically challenging [2, 3]. Meanwhile, sickness absence due to mental ill-health and pain has increased, especially among younger employees [4, 5].

Work disability is associated with a significant economic loss, for both society, employers, and individuals. Labor market cost includes both presenteeism and absenteeism [6]. Besides working as ill, presenteeism also captures and can be defined as productivity loss related to reduced capacity to work due to health problems [6, 7]. The cost of presenteeism is difficult to measure, but it may even exceed the cost of sickness absence [7–9]. This is highly relevant for Finnish employers, who bear the full cost of presenteeism and the first 1–10 sickness absence days, which account for over 70% of sickness absence costs among municipal employees under 35. After 10 days, costs are partly covered by the Social Insurance Institution of Finland. In 2021, public expenditure on disability and sickness cash benefits averaged 2.0% of gross domestic product (GDP) across Organisation for Economic Co-operation and Development (OECD) countries and 3.1% in Finland, reflecting the issue’s also broader societal relevance [10].

Besides legal responsibilities, employers have a strong economic incentive to support employees’ work ability, which is crucial for productivity, organizational success, and societal prosperity [2]. Therefore, understanding working conditions’ contribution to labor market costs and the potential economic benefits of improving them is important. A review of occupational health interventions showed a non-significant reduction in sickness absence but a positive return on investment, possibly due to unmeasured improvements in presenteeism [11]. A Japanese quasi-experimental study found associations between employee exercise habits and work engagement, but not presenteeism [12]. A Danish study showed that simulated improvements in influence at work, such as greater control over work schedules and task prioritization, may reduce sickness absence [13]. Overall, previous research has focused more on health behaviors and sickness absence.

Although working conditions are associated with sickness absence [13–19] and often also presenteeism [20, 21], and employers can influence working conditions more than individual health behaviors, few studies have examined the associated labor market cost. A Danish study estimated annual absenteeism losses due to work-related stress at €1903 for men and €3909 for women [22]. However, it did not consider presenteeism, total employer’s cost, or working conditions beyond stress. To our knowledge, no previous studies have quantified employer’s cost related to working conditions while accounting for both presenteeism and sickness absence. Making the cost visible may encourage employers to improve working conditions and enable earlier, more targeted action on factors most strongly associated with costs, potentially before sickness absence occurs or employees recognize a decline in work ability.

Therefore, this register-linked study aimed to examine how workplace atmosphere, workplace bullying, social support, organizational justice, work-family conflict, and physical working conditions are associated with employer’s cost of presenteeism, sickness absence of first 1–10 days, and subsequent 11–60 days among young and early midlife municipal employees. As marital status and education are associated with both working conditions and work ability [23, 24], we included these as covariates.

## Materials and methods

### Participants

**Fig. 1:**
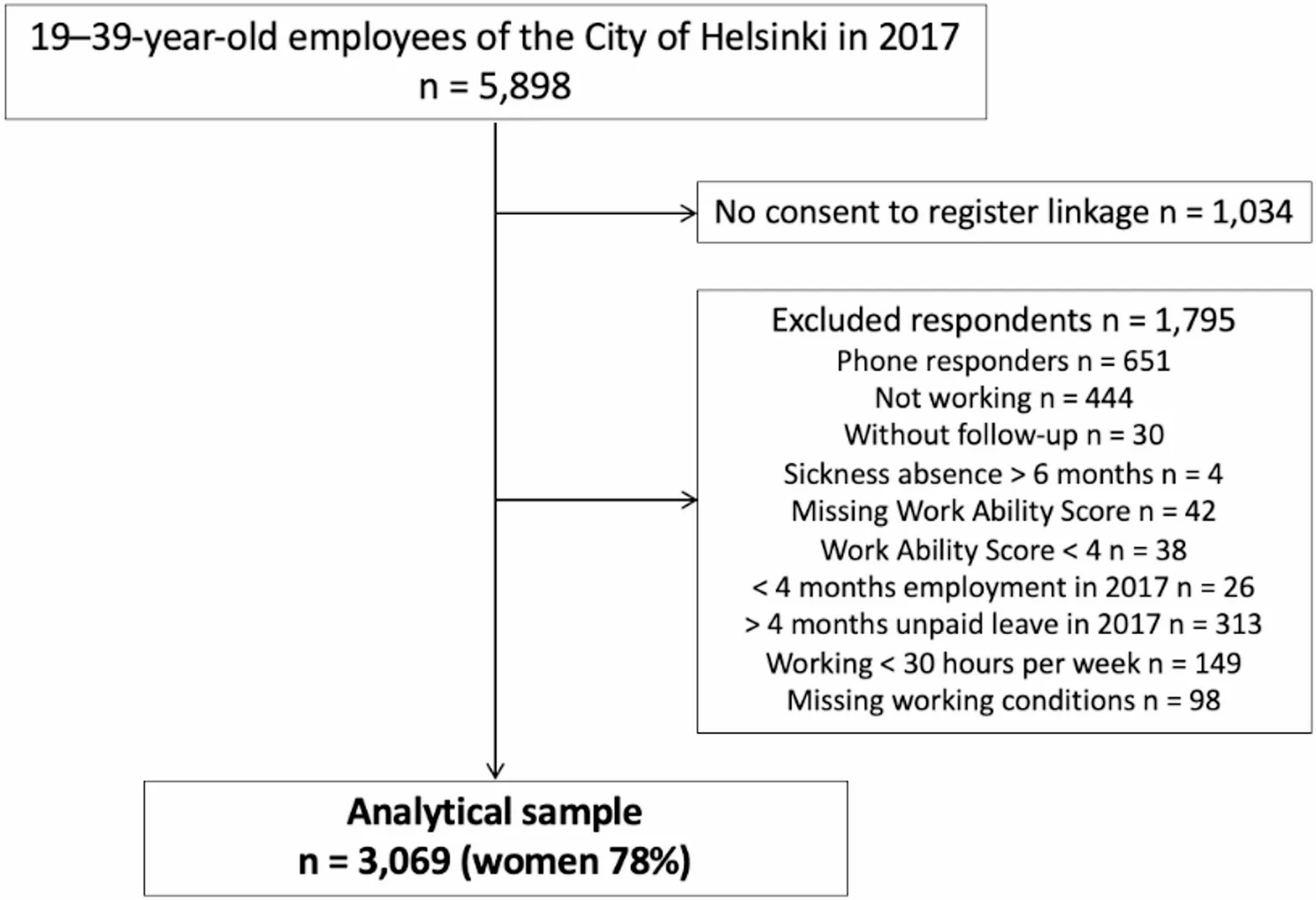
Flowchart of the study population selection

The Helsinki Health Study is an ongoing cohort study including employees of the City of Helsinki, which is the largest employer in Finland with around 38,000 employees representing a wide range of occupations and sectors [25]. Data were collected via online and postal questionnaires among 19–39-year-old employees with at least a 50% employment contract for at least 4 months before the start of the data collection in 2017 (target population *n* = 11,459). Additionally, a short telephone interview (*n* = 787) was conducted among those who did not respond to the online or postal questionnaire. In total, 5,898 participants responded, corresponding to a response rate of 51.5%. Of these, 4,864 participants (82%) consented to register linkage (Fig. 1). The majority of respondents were women (78%), reflecting the gender distribution of the target population and the municipal sector in Finland [25].

We excluded phone respondents (*n* = 651) because the interview lacked information on all key variables. We also excluded participants not working full- or part-time at baseline (*n* = 444), without follow-up data (*n* = 30), or with sickness absence exceeding 6 months during follow-up (*n* = 4). Participants with missing Work Ability Score (WAS) (*n* = 42) or a WAS < 4 (*n* = 38), were excluded, because low WAS is associated with high sickness absence and may overestimate presenteeism [26]. We further excluded participants with unpaid leave of > 4 months (*n* = 313), employment contract lasting < 4 months in 2017 (*n* = 26), and or working < 30 h/week (*n* = 149). After excluding those with missing working conditions (*n* = 98) the final dataset included 2,399 women and 670 men.

### Exposure: working conditions

#### Workplace atmosphere

We asked the participants about the workplace atmosphere with the following question: “What kind of atmosphere do you have at your current workplace?” Response options ranged from “very good” to “very bad” and were categorized as good (“very good” or “good”), intermediate (“satisfactory”), and bad (“bad” or “very bad”).

#### Workplace bullying

Workplace bullying was described as follows: “Psychological violence or bullying at work refers to a constant, repeated isolation of a member of the work community, belittling one’s work effort, threats, talking behind one’s back or other forms of pressure.” We then asked: “Do you think such behavior takes place at your work unit?” and “Have you suffered from such bullying?”. Response options for the first question were “not at all”, “sometimes”, “constantly”, and “cannot say”; for the second, “no”, “yes, currently”, “yes, earlier in this job”, “yes, earlier in another job”, and “cannot say”. Participants who reported suffering from bullying earlier were combined [27].

#### Social support

We measured social support from colleagues and immediate supervisor using two questions for each from the Copenhagen Psychosocial Questionnaire [28]: “How often do you get help and support from your colleagues/immediate supervisor?” and “How often are your colleagues/immediate supervisor willing to listen to your work-related problems?”. There were four response options per question (“often”, “sometimes”, “seldom”, “never”), scored from 1 to 4. We summed the scores for social support from colleagues and from supervisors, and participants were classified as having high perceived social support from colleagues and supervisor (2 points), average support from colleagues (3 points) and supervisors (3–4 points), and high support from colleagues (4–8 points) and supervisors (5–8 points).

#### Organizational justice

We measured organizational justice with a shorter version of Moorman’s inventory [29], using four questions on procedural justice and four on relational justice. Relational justice perceived fairness of supervisor behavior: (i) “My supervisor listens to his/her employees’ opinions regarding important matters”; (ii) “My supervisor’s personal views do not have an adverse effect on his/her decisions”; (iii) “My supervisor treats his/her employees with kindness and consideration”; (iv) “I can trust my supervisor”. Procedural justice measured fairness of organizational decision-making processes: (i) “In my work community, decisions are made based on accurate information”; (ii) “Everyone has the right to express their own opinions on matters affecting them”; (iii) “Decisions made in my work community are consistent (the same rules apply to everyone)”; (iv) “In my work community, decisions that prove ineffective or counter-productive can be reversed or changed”. Questions were answered on a five-point scale ranging from “Completely agree” to “Completely disagree”. Sum scores [4–9, 11–13, 20] were calculated for relational and procedural justice and categorized into low, intermediate, and high groups based on tertiles.

#### Work–family conflicts

We used a questionnaire adapted from the National Study of Midlife Development in the USA to measure work-family conflicts [30], assessing the extent to which job responsibilities interfere with family life. The questions were “Your job reduces the amount of time you can spend with the family”, “Problems at work make you irritable at home”, “Your job involves a lot of travel away from home”, and “Your job takes so much energy you don’t feel up to doing things that need attention at home”. There were four response options (“Not at all”, “To some extent”, “A great deal”, “Not Applicable”), coded from 1 to 4. We formed four groups: no conflicts (score 4), intermediate conflicts (scores 5–7), high conflicts (scores 8–12), and those with not applicable (with at least one item marked as “not applicable”, e.g. no family) [31].

#### Physical working conditions

We used factor analysis of an 18-item questionnaire battery developed at the Finnish Institute of Occupational Health to measure different physical work exposures [32]. The three factors were: (1) hazardous exposure (e.g. exposure to chemicals, noise, or dust and dirt) (2) physical workload (e.g. repetitive movements, uncomfortable postures, lifting, or walking), and (3) desktop work (e.g. computer use and sedentary work) [33]. Each measure was classified into three groups according to the lowest and highest tertile cut-points. 

### Outcome measures: employer’s cost related to presenteeism and sickness absence

We received information on sickness absence, employment contract, and parental leave from the City of Helsinki personnel register. Follow-up for sickness absence started the day after receiving the questionnaire and continued for one year or until the end of employment, whichever came first. Follow-up time was adjusted by excluding paid and unpaid parental leave, during which sickness absence is unusual. We calculated the total sickness absence days during the follow-up, including also self-certified sickness absence, which can be authorized by supervisors for up to one week without a medical certificate.

To estimate the annual presenteeism days lost due to reduced work ability, we used the baseline WAS, which is a person’s estimate of one’s current work ability from 0 to 10, compared to one’s lifetime best [26]. In theory, a one-point decrease below 9 on the WAS is associated with an approximately 5% productivity loss [26]. First, we defined the number of annual working days as 227, based on the average in Finland. Next, we adjusted this figure according to each individual’s follow-up time and subtracted their sickness absence days to obtain annual net working days. Finally, we multiplied annual net working days by WAS-related discount factors to estimate annual number of presenteeism days. Both sickness absence and presenteeism were standardized to person-years (mean follow-up 0.90 years) for analysis.

We used individual annual gross salary data from 2017, converted to 2025 earnings level (third quartile) using the Statistics Finland wage and salary index for local government employees [34]. The index values averaged 99.9 for women and 98.9 for men in 2017, and 133.3 for women and 126.8 for men in the 2025 fourth quartile. We divided the participants’ annual salary by the participants’ number of working days in 2017, excluding unpaid parental leave, to estimate the average cost of one working day. Extremely low daily costs were truncated to a minimum of €100 per day for full-time work for a very small number of participants (*n* = 5). We multiplied annual presenteeism and sickness absence days with the cost of one working day. For 11–60 days sickness absence, we adjusted the daily cost to 30%, which reflects the employer’s cost after the Social Insurance Institution of Finland reimburses about 70%.

### Covariates

Covariates were derived from the survey. We used three age groups: 19–29, 30–34, and 35–39 years and classified gender as women and men, marital status as married/cohabiting and others (single, divorced, or widowed), and education as upper secondary school or less, bachelor’s degree, and master’s degree or higher.

Missing information on marital status (*n* = 6) or education (*n* = 3) was negligible, and thus the missing responses were classified as single and having low education, respectively, to avoid unnecessary exclusions [35].

### Statistical analyses

First, we described the distribution of baseline characteristics among women and men using cross-tabulation for categorical variables. Additionally, we calculated means (standard deviations, SDs) and medians (interquartile ranges, IQRs) for daily costs, presenteeism, and sickness absence days standardized to one person-year during the subsequent one-year follow-up.

Second, we used a two-part model (“twopartm” package in R) to analyze associations between demanding working conditions and employer’s cost (€) related to presenteeism and sickness absence (1–10 days and 11–60 days). The first part used logistic regression to estimate the probability of having non-zero outcomes, and the second part used a generalized linear model with a gamma distribution to examine employer’s cost among those with positive values. We then obtained individual-level estimated cost using the model’s prediction function and calculated the mean cost as the average of these predicted values to estimate employer’s cost of presenteeism and sickness absence per 10 person-years by exposure to demanding working conditions [35]. Finally, we calculated marginal effects with 95% confidence intervals (CI) to compare on employer’s annual cost (standardized to 1 person-year) of presenteeism and sickness absence by exposure to working conditions. Model 1 adjusted for age, and Model 2 additionally for marital status and education. Participants without demanding working conditions served as reference groups. We analyzed women and men separately, because it is known that the associations between working conditions, presenteeism and sickness absence differ by gender [35–37]. This is partly due to differences in working conditions and exposures between women and men, and gender-specific results are important for developing targeted recommendations.

We used IBM SPSS Statistics 31 for the descriptive analyses, and RStudio for estimating costs and calculating the mean effects using the two-step model.

## Results

During the follow-up year (mean follow-up 0.9 years, standardized to 1 person-year) women had an estimated average of 11.4 and men 10.6 presenteeism days (Table 1). Women seemed to have more sickness absence days than men, with a mean of 11.1 vs. 6.9 days for 1–10 days and 2.8 vs. 1.4 days for 11–60 days. Men had a slightly higher salary and the average cost per working day was €213 for women and €222 for men. Overall, mean annual employer’s cost of presenteeism, sickness absence of 1–10 days, and 11–60 days were €2,395, €2,229, and €541 for women, and €2,245, €1,422, and €306 for men, respectively.

**Table 1:** Baseline characteristics at Phase 1 in 2017 and presenteeism and sickness absence days during the subsequent one-year follow-up among young and early midlife female (*n* = 2,399) and male (*n* = 670) employees of the City of Helsinki

Characteristics at Phase 1	Women	Men
*Total*,* n*	2399	670
*Age*,* n (%)*		
19–29	861 (36)	183 (27)
30–34	788 (33)	214 (32)
35–39	750 (31)	273 (41)
*Marital status*,* n (%)*		
Married/cohabiting	1519 (63)	489 (73)
Others	880 (37)	181 (27)
*Education*,* n (%)*		
Upper secondary school	754 (31)	293 (44)
Bachelor’s degree	930 (38)	194 (29)
Master’s degree or higher	715 (30)	183 (27)
Follow-up time in days		
Mean (SD)	326 (87)	341 (66)
Median (IQR)	364 (0)	364 (0)
Average cost (€) / working day		
Mean (SD)	213 (67)	222 (68)
Median (IQR)	196 (66.7)	201 (62.3)
Presenteeism days^1^		
Mean (SD)	11.4 (12.8)	10.6 (13.3)
Median (IQR)	11 (20)	10 (11.6)
Sickness absence 1–10 days^1^		
Mean (SD)	11.1 (15.4)	6.9 (9.2)
Median (IQR)	6 (12)	3 (10)
Sickness absence 11–60 days^1^		
Mean (SD)	2.8 (13.9)	1.4 (7)
Median (IQR)	0 (0)	0 (0)

Abbreviations: SD= standard deviation; IQR = interquartile range

^1^ Follow-up standardized to one person-year per participant (mean follow-up 0.9 years)

**Fig. 2:**
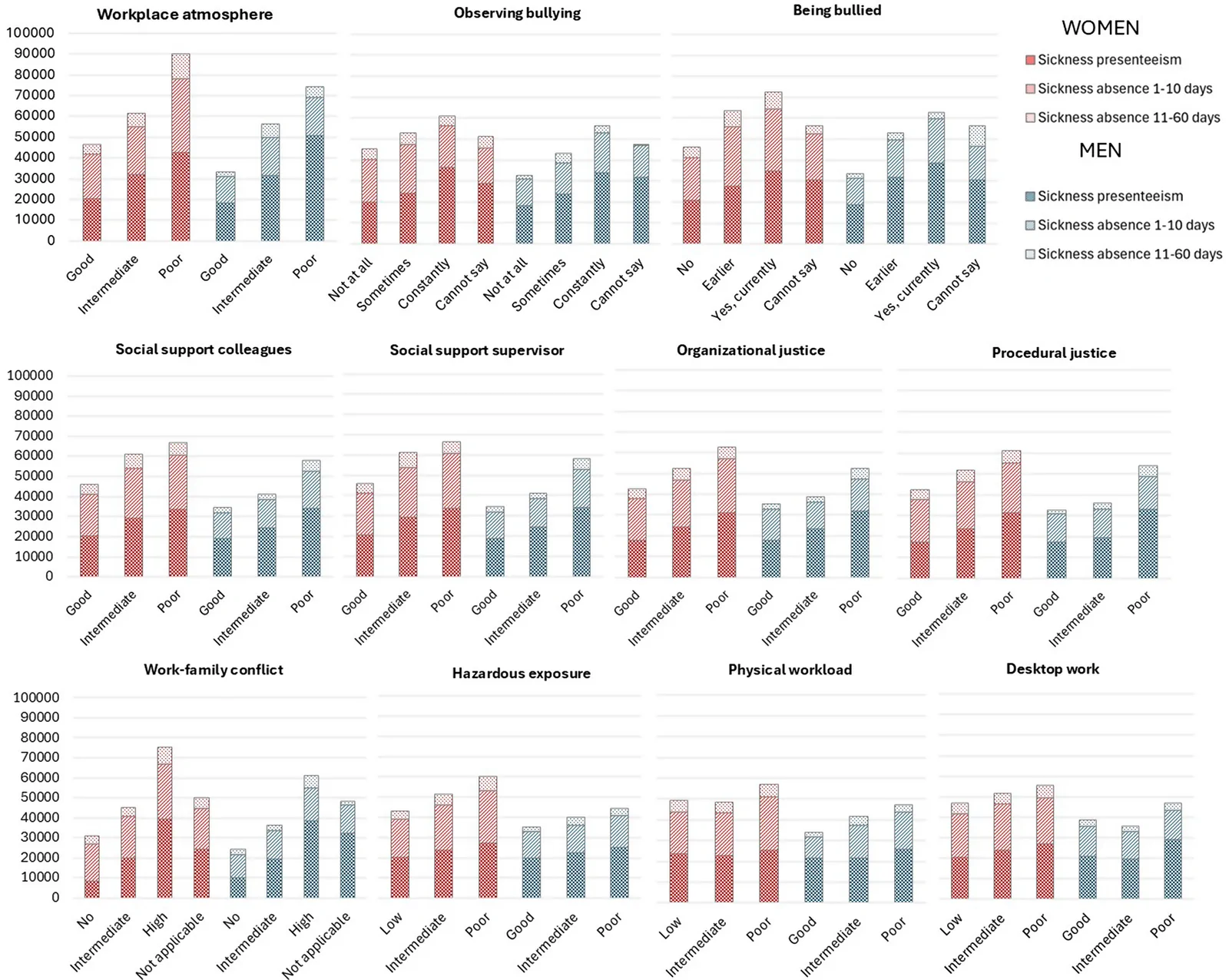
Age-adjusted employer’s cost (€) of presenteeism and sickness absence during one-year follow-up per 10 person-years by psychosocial and physical working conditions in 2017 among young and early midlife female (*n* = 2,399) and male (*n* = 670) employees of the City of Helsinki, Finland

Figure 2 presents age-adjusted estimated cost for presenteeism and sickness absence (1–10 and 11–60 days) per 10 person-years by psychosocial and physical working conditions among women and men. Women and men with psychosocially demanding working conditions seemed to have higher estimated cost of presenteeism, suggesting that the cost is increasing with increasing work demands, particularly for workplace atmosphere and work-family conflict. Similar findings were found for sickness absence, especially for 1–10 days. However, for physical working conditions, the differences were overall less pronounced.

The employer’s cost of presenteeism were higher among women reporting demanding working conditions, whereas among men, the cost was similar but statistically weaker (Tables 2 and 3). Especially those with high work-family conflicts (women: €3,108, 95% CI €2,762–3,454; men €2,701, 95% CI €1,993–3,409) and poor workplace atmosphere (women: €2,203, 95% CI €1,443–2,962; men €3,136, 95% CI €1,219–5,053) had higher age-adjusted presenteeism cost compared to those without these exposures. Also women and men reporting workplace bullying, poor social support, and low organizational justice had higher employer’s cost of presenteeism. The differences in cost of sickness absence of 1–10 days were similar but somewhat weaker than for presenteeism, especially among men (for example, high work-family conflicts among women; €935, 95% CI €462–1,407 and men; €539, 95% CI €84–995). For physical working conditions, hazardous exposure was associated with higher presenteeism and sickness absence cost among women, whereas physical workload was associated with higher cost of sickness absence of 1–10 days and desktop work with higher presenteeism cost among both women and men. Differences in the cost of sickness absence of 11–60 days were statistically non-significant among both women and men, except for workplace bullying among men and hazardous exposure among women. After adjustments for education and marital status, the association between physical workload and the cost of sickness absence of 1–10 days reduced among women, but strengthened for presenteeism among both genders (Tables 4 and 5). Among men, adjustments for education and marital status attenuated the associations between workplace bullying and the cost of sickness absence of 1–10 days. Otherwise, the associations remained similar in the fully adjusted model.

**Table 2:** Age-adjusted marginal effects and their 95% confidence intervals (95% CI) on employer’s annual cost (€; standardized to 1 person-year) of presenteeism and sickness absence (first 1–10 and subsequent 11–60 days) among young and early midlife female (*n* = 2,399) employees of the City of Helsinki, Finland, by demanding working conditions in 2017

		WOMEN					
		Presenteeism cost (€)		Sickness absence 1–10 days cost (€)		Sickness absence 11–60 days cost (€)	
Exposure group	n	dy/dx	95% CI	dy/dx	95% CI	dy/dx	95% CI
*Workplace atmosphere*							
Good	1763	ref.		ref.		ref.	
Intermediate	548	1167	868–1466	180	−124–484	127	−185–439
Poor	88	2203	1443–2962	1473	412–2532	722	−384–1828
*Observing workplace bullying*							
Not at all	855	ref.		ref.		ref.	
Sometimes	1225	406	168–644	302	33–571	69	−188–325
Constantly	194	1645	1098–2192	951	358–1544	−39	−408–329
Cannot say	125	858	317–1399	−292	−769–184	7	−534–549
*Being bullied at work*							
No	1576	ref.		ref.		ref.	
Earlier	416	663	345–982	845	441–1249	220	−105–544
Yes, currently	96	1381	702–2060	984	152–1816	287	−464–1038
Cannot say	311	938	560–1316	188	−181–557	−110	−357–136
*Social support colleagues*							
Good	1636	ref.		ref.		ref.	
Intermediate	357	870	529–1211	421	34–807	256	−133–646
Poor	406	1325	982–1668	637	250–1023	130	−194–454
*Social support supervisor*							
Good	854	ref.		ref.		ref.	
Intermediate	1060	893	654–1131	214	−68–497	−46	−311–219
Poor	485	1487	1155–1818	296	−63–655	−75	−385–235
*Interactional justice*							
Good	863	ref.		ref.		ref.	
Intermediate	843	627	373–881	289	−2–581	65	−210–340
Poor	693	1313	1021–1605	502	182–822	89	−198–375
*Procedural justice*							
Good	805	ref.		ref.		ref.	
Intermediate	836	623	373–873	247	−48–543	69	−205–343
Poor	758	1385	1095–1674	420	101–739	115	−170–400
*Work-family conflict*							
Low	223	ref.		ref.		ref.	
Intermediate	1407	1183	941–1425	243	−149–635	−3	−349–344
High	586	3108	2762–3454	935	462–1407	396	−44–836
Not applicable	183	1596	1152–2040	175	−392–742	127	−379–632
*Hazardous exposure*							
Low	751	ref.		ref.		ref.	
Intermediate	846	334	60–608	343	53–633	139	−122–400
High	802	688	408–968	739	423–1055	292	10–574
*Physical workload*							
Low	768	ref.		ref.		ref.	
Intermediate	785	−47	−345–251	−1	−302–300	−74	−360–211
High	846	184	−104–472	490	165–815	29	−258–316
*Desktop work*							
Low	712	ref.		ref.		ref.	
Intermediate	818	339	68–610	128	−182–438	7	−279–292
High	869	644	361–926	163	−151–477	79	−227–386

**Table 3:** Age-adjusted marginal effects and their 95% confidence intervals (95% CI) on employer’s annual cost (€; standardized to 1 person-year) of presenteeism and sickness absence (first 1–10 and subsequent 11–60 days) among young and early midlife male (n = 670) employees of the City of Helsinki, Finland, by demanding working conditions in 2017

		MEN						
		Presenteeism cost (€)			Sickness absence 1–10 days cost (€)		Sickness absence 11–60 days cost (€)	
Exposure group	n	dy/dx	95% CI		dy/dx	95% CI	dy/dx	95% CI
Workplace atmosphere								
Good	497	ref.			ref.		ref.	
Intermediate	148	1257	707–1807		583	190–977	380	−38–799
Poor	25	3136	1219–5053		564	−404–1532	294	−394–982
*Observing workplace bullying*								
Not at all	295	ref.			ref.		ref.	
Sometimes	290	521	64–977		197	−99–494	258	−12–529
Constantly	51	1477	548–2406		644	11–1278	145	−267–556
Cannot say	34	1477	284–2671		201	−419–820	−121	−299–58
Being bullied at work								
No	481	ref.			ref.		ref.	
Earlier	71	1206	459–1953		485	−50–1021	130	−175–436
Yes, currently	31	1800	666–2934		825	−84–1734	100	−281–482
Cannot say	87	1162	430–1895		347	−87–781	749	31–1467
Social support colleagues								
Good	432	ref.			ref.		ref.	
Intermediate	125	453	−121–1027		131	−221–484	8	−325–341
Poor	113	1442	758–2127		573	113–1033	247	−186–680
Social support supervisor								
Good	297	ref.			ref.		ref.	
Intermediate	269	712	259–1166		225	−84–535	216	−64–496
Poor	104	1433	694–2173		53	−339–444	47	−212–306
Interactional justice								
Good	320	ref.			ref.		ref.	
Intermediate	219	494	26–961		−190	−502–122	13	−257–283
Poor	131	1306	643–1969		59	−328–446	266	−134–666
Procedural justice								
Good	272	ref.			ref.		ref.	
Intermediate	209	97	−363–557		49	−265–364	86	−142–313
Poor	189	1470	893–2047		219	−144–583	356	−4–717
Work-family conflict								
Low	127	ref.			ref.		ref.	
Intermediate	362	892	468–1316		300	−33–633	−40	−319–238
High	138	2701	1993–3409		539	84–995	298	−198–794
Not applicable	43	2318	1262–3374		283	−338–904	−125	−470–221
Hazardous exposure								
Low	240	ref.			ref.		ref.	
Intermediate	185	299	−242–839		32	−305–369	201	−167–569
High	245	484	−36–1003		242	−94–579	151	−114–416
Physical workload								
Low	262	ref.			ref.		ref.	
Intermediate	247	36	−454–525		553	241–864	254	−84–593
High	161	508	−95–1111		760	371–1148	181	−99–462
Desktop work								
Low	308	ref.			ref.		ref.	
Intermediate	200	−85	−593–422		−84	−410–243	−69	−356–219
High	162	737	167–1307		−39	−297–318	38	−295–371

**Table 4:** Marginal effects and their 95% confidence intervals (95% CI) on employer’s annual cost (€; standardized to 1 person-year) of presenteeism and sickness absence (first 1–10 and subsequent 11–60 days) among young and early midlife female (*n* = 2,399) employees of the City of Helsinki, Finland, by demanding working conditions in 2017. Adjusted for age, marital status, and education

		WOMEN					
		Presenteeism cost (€)		Sickness absence 1–10 days cost (€)		Sickness absence 11–60 days cost (€)	
Exposure group	n	dy/dx	95% CI	dy/dx	95% CI	dy/dx	95% CI
*Workplace atmosphere*							
Good	1763	ref.		ref.		ref.	
Intermediate	548	1143	851–1435	162	−142–467	146	−157–449
Poor	88	2227	1483–2972	1448	386–2510	646	−337–1629
*Observing workplace bullying*							
Not at all	855	ref.		ref.		ref.	
Sometimes	1225	487	254–720	276	5–547	30	−222–282
Constantly	194	1762	1211–2313	887	294–1479	−74	−430–283
Cannot say	125	856	337–1376	−305	−785–174	−41	−544–462
*Being bullied at work*							
No	1576	ref.		ref.		ref.	
Earlier	416	752	430–1075	775	370–1179	166	−143–475
Yes, currently	96	1516	830–2202	925	96–1754	182	−475–839
Cannot say	311	1011	633–1390	133	−237–503	−137	−379–105
*Social support colleagues*							
Good	1636	ref.		ref.		ref.	
Intermediate	357	826	498–1154	425	34–815	259	−116–635
Poor	406	1408	1060–1756	571	185–956	98	−206–402
*Social support supervisor*							
Good	854	ref.		ref.		ref.	
Intermediate	1060	920	687–1154	219	−65–504	−30	−287–228
Poor	485	1503	1176–1829	240	−118–599	−69	−372–233
*Interactional justice*							
Good	863	ref.		ref.		ref.	
Intermediate	843	629	383–876	280	−14–574	27	−238–293
Poor	693	1400	1110–1690	453	132–774	62	−218–341
*Procedural justice*							
Good	805	ref.		ref.		ref.	
Intermediate	836	629	387–876	251	−46–548	60	−200–320
Poor	758	1408	1125–1691	423	103–744	133	−144–411
*Work-family conflict*							
Low	223	ref.		ref.		ref.	
Intermediate	1407	1214	976–1452	262	−133–657	5	−339–349
High	586	3169	2825–3514	938	460–1415	348	−77–773
Not applicable	183	1498	1060–1936	175	−402–751	175	−364–714
*Hazardous exposure*							
Low	751	ref.		ref.		ref.	
Intermediate	846	305	38–572	336	44–628	153	−104–411
High	802	738	459–1018	699	381–1017	257	−8–522
*Physical workload*							
Low	768	ref.		ref.		ref.	
Intermediate	785	30	−255–316	−60	−371–251	−148	−466–171
High	846	514	200–829	352	−3–708	−110	−433–213
*Desktop work*							
Low	712	ref.		ref.		ref.	
Intermediate	818	352	80–624	165	−143–473	−11	−270–249
High	869	614	328–901	322	−7–650	154	−157–466

**Table 5:** Marginal effects and their 95% confidence intervals (95% CI) on employer’s annual cost (€; standardized to 1 person-year) of presenteeism and sickness absence (first 1–10 and subsequent 11–60 days) among young and early midlife male (*n* = 670) employees of the City of Helsinki, Finland, by demanding working conditions in 2017. Adjusted for age, marital status, and education

		MEN					
		Presenteeism cost (€)		Sickness absence 1–10 days cost (€)		Sickness absence 11–60 days cost (€)	
Exposure group	n	dy/dx	95% CI	dy/dx	95% CI	dy/dx	95% CI
*Workplace atmosphere*							
Good	497	ref.		ref.		ref.	
Intermediate	148	1298	741–1855	595	194–996	444	−35–924
Poor	25	3259	1304–5214	537	−433–1506	333	−424–1089
*Observing workplace bullying*							
Not at all	295	ref.		ref.		ref.	
Sometimes	290	565	101–1028	148	−156–452	228	−42–499
Constantly	51	1555	584–2526	496	−132–1123	109	−300–519
Cannot say	34	1437	260–2613	170	−460–799	−131	−324–61
*Being bullied at work*							
No	481	ref.		ref.		ref.	
Earlier	71	1287	526–2048	522	−37–1082	131	−194–456
Yes, currently	31	1944	740–3148	634	−249–1517	67	−297–432
Cannot say	87	1225	475–1976	258	−171–687	736	−7–1479
*Social support colleagues*							
Good	432	ref.		ref.		ref.	
Intermediate	125	480	−97–1056	127	−230–484	15	−345–374
Poor	113	1476	783–2168	597	123–1070	175	−221–571
*Social support supervisor*							
Good	297	ref.		ref.		ref.	
Intermediate	269	727	273–1181	289	−26–604	267	−56–590
Poor	104	1435	686–2184	50	−340–440	62	−212–336
*Interactional justice*							
Good	320	ref.		ref.		ref.	
Intermediate	219	447	−16–910	−196	−515–123	−2	−267–263
Poor	131	1406	720–2092	−2	−390–386	281	−139–701
*Procedural justice*							
Good	272	ref.		ref.		ref.	
Intermediate	209	125	−330–579	82	−234–398	95	−132–322
Poor	189	1550	967–2133	247	−120–613	387	−17–791
*Work-family conflict*							
Low	127	ref.		ref.		ref.	
Intermediate	362	1039	624–1455	289	−51–629	−60	−365–245
High	138	2929	2210–3647	578	107–1049	344	−221–909
Not applicable	43	1842	845–2840	288	−369–944	−154	−515–207
*Hazardous exposure*							
Low	240	ref.		ref.		ref.	
Intermediate	185	306	−223–835	14	−339–366	233	−149–614
High	245	585	40–1131	83	−266–432	140	−120–399
*Physical workload*							
Low	262	ref.		ref.		ref.	
Intermediate	247	52	−453–557	454	122–785	266	−98–630
High	161	709	−26–1393	579	163–996	153	−159–466
*Desktop work*							
Low	308	ref.		ref.		ref.	
Intermediate	200	−46	−555–464	−74	−403–255	−66	−355–223
High	162	790	209–1370	58	−323–438	39	−292–370

## Discussion

The main findings showed that psychosocially demanding working conditions were associated with higher employer’s cost of presenteeism and first 1–10 sickness absence days among young and early midlife employees. Work–family conflict, poor workplace atmosphere, workplace bullying, low social support, low organizational justice, and desktop work were associated with higher presenteeism cost. Annual presenteeism cost was up to €3,000 higher among those reporting psychosocially demanding working conditions. Similar associations were observed for the employer’s cost of sickness absence of 1–10 days, with cost up to €1,000 higher among women with demanding working conditions. The associations were statistically weaker among men. For longer sickness absence of 11–60 days, associations with employer’s cost were weak in both genders after accounting for reimbursement (70%). However, longer sickness absence imposes substantial cost on society (sickness absence benefits almost €1 billion in Finland in 2025) and is associated with other indirect employer’s cost related to, for example, recruitment, replacement staffing, and productivity losses. For physically demanding working conditions, hazardous exposure among women was associated with higher cost of presenteeism and physical workload was associated with higher age-adjusted cost of sickness absence of 1–10 days among both genders.

Our results align with previous studies showing that demanding working conditions are associated with presenteeism [20, 36] and sickness absence [14, 15, 17, 18]. In our study, the association between psychosocially demanding working conditions and presenteeism cost was stronger than for sickness absence cost, whereas physical workload was stronger associated with age-adjusted sickness absence cost. This is consistent with findings from a previous study using the same cohort, which showed that perceived mental strenuousness of work was strongly associated with presenteeism and weaker with sickness absence [37]. In contrast, physically demanding work was associated with sickness absence and weaker with presenteeism. However, the study did not consider costs or broader working conditions.

After adjustments for education and marital status, the association between physical workload and the cost of sickness absence of 1–10 days lost statistical significance among women, but strengthened for presenteeism among both genders. Physically demanding work is more common among lower-educated employees, who also have higher sickness absence rates compared with higher-educated employees [23]. This may reflect difficulties in performing physical tasks when ill, but may also be related to other factors associated with lower education. In contrast, highly educated employees may have higher threshold for sickness absence and may continue working while ill, for example by working remotely during mild illnesses, thereby increasing presenteeism.

However, there are few previous studies on employer’s cost, especially considering both presenteeism and sickness absence. A Danish study estimated that overall work-related stress was associated with an annual work absenteeism loss of €3909 for women and €1903 for men, with higher cost among employees aged over 35 than younger [22]. Of the cost of work absenteeism, 60% for men and 68% for women consisted of wage payments during sickness absence, but direct employer’s cost was not specified and presenteeism was not considered. However, making monetary comparisons between countries is challenging, due to differences in wages, insurance systems, and cultural factors associated with presenteeism and sickness absence [38]. Measuring presenteeism cost is even more challenging, and it may exceed sickness absence cost [7–9]. In Japan, estimated presenteeism cost per 1,000 employees were $488,210 for low back pain, $346,308 for neck pain or stiff shoulders, and $327,137 for mental illness [39], whereas in a Finnish private-sector company, presenteeism cost accounted for approximately 3.7% of personnel cost, with €1,302 annual cost per employee in 2008 [40]. The Finnish study used the same WAS-based method, reporting 8.7 to 10.4 annual presenteeism days per employee, which is similar to our findings (11.4 days for women and 10.6 for men). Overall sickness absence was more common among women than men in our study, whereas the difference in presenteeism days was small.

To our knowledge, this is the first study examining the association of working conditions with the employer’s cost of both presenteeism and sickness absence among young and early midlife employees. Several psychosocially demanding working conditions were associated with substantial employer’s costs. Making these costs visible is important and may encourage employers to invest in improving working conditions and target those most strongly associated with cost. Such investments may not only support productivity and employee well-being but also, more broadly, strengthen the basis of successful and sustainable societies [2]. In our study the association between psychosocial working conditions and employer’s cost of presenteeism were stronger than for sickness absence, highlighting the need for more studies especially on presenteeism. This could enable earlier preventive actions, even before sickness absence occurs or employees recognize the decline themselves.

### Methodological considerations

It is important to acknowledge some limitations. First, the response rate in the survey was acceptable (51.5%), but non-response and potential healthy worker bias may still exist, as individuals with a lower socioeconomic position and a long-term sickness absence, both before or during data collection, were somewhat less likely to respond [25].

Second, working conditions and covariates was self-reported. However, we included several measures of both psychosocial and physical working conditions, and objective data on these are rarely available. Additionally, objective measures may not fully capture perceived working conditions, which are relevant for work ability.

Third, although sickness absence and individual annual gross salary were based on reliable register data, presenteeism was estimated indirectly using a WAS-based measurement tool developed by Vänni et al. and validated in two Finnish industrial sectors (food and forest industries) [26]. The scale assumes that each one-point decrease below 9 on the WAS is associated with an approximate 5% productivity loss. The baseline WAS combined with follow-up data on sickness absence and working days, allows the estimation of annual presenteeism days during the follow-up.

The method has not been validated in the public sector studied here, but the annual presenteeism days were similar to those in the original validation study [26]. In the absence of direct productivity measures, this provides a practical proxy for estimating productivity loss due to presenteeism. This measure is also similar to the Single-Item Presenteeism Question derived from the WHO’s Health and Work Performance Questionnaire used in other studies [12]. A common alternative is self-reported days worked while ill [36], but this captures only frequency of working while ill and not the severity of impairment or its impact on work ability. Presenteeism refers to reduced productivity while at work due to health-related or other factors and is associated not only with ill-health, but also with adverse organizational factors, lower job satisfaction, reduced work engagement, and emotional exhaustion [8].

Fourth, the cohort consisted of Finnish public sector employees, mainly from the capital region. Although a wide range of occupations and occupational social classes was represented, generalizability might be limited, particularly regarding men. In line with the gender distribution in the public sector, most participants were women, and we may lack statistical power for strong gender-specific conclusions. Additionally, working conditions and wages differ to some extent from those in the private sector, and productivity losses in the public sector are more difficult to interpret, as productivity is less easily quantified in monetary terms. We did not use a wage multiplier to account for employer-related social cost, but these are estimated to be at least 20% in Finland [41]. There are also other direct and indirect employer’s cost, such as hiring substitutes, reduced efficiency, and production delays, but these could not be included.

Nevertheless, the large cohort combined with reliable register-based data enabled us to make visible the employer’s cost of presenteeism and sickness absence associated with working conditions among young and early midlife employees. Further studies in different cultural settings and sectors are needed, as employee well-being is a crucial determinant of productivity and, more broadly, the basis of successful companies and sustainable societies.

## Conclusions

Work–family conflict, poor workplace atmosphere, workplace bullying, low social support, and organizational injustice were associated with higher employer’s cost of especially presenteeism, but also sickness absence. Displaying costs of early declines in work ability encourages improvements in working conditions and timely, targeted preventive actions, benefiting both employees and employers, as well as generating broader societal and economic benefits.

## Data Availability

Data are not publicly available due to strict data protection laws and can only be accessed by the members of the study group (https://www.helsinki.fi/en/researchgroups/helsinki-health-study/data-protection-statement). Further inquiries can be directed to the corresponding author.

## Abbreviations

CI: Confidence interval
GDP: Gross domestic product
IQR: Interquartile range
OECD: Organization for economic co-operation and development
SD: Standard deviation
WAS: Work ability score
